# Preventive Medicine

**Published:** 1906-01

**Authors:** 


### PREVENTIVE MEDICINE.

TO HIM who is tinged with therapeutic infidelity aud descants upon the incompetence of drugs as measured against disease, the achievements of preventive medicine should bring some sunshine into his cynicism. It has been the fashion among some practitioners to belittle the strength of drug therapy and to point as substantiation for their lack of faith, to the small parcel of specifics which have been taken from the vast stock of materia medica and which undoubtedly looks pitiably meager and insignificant beside the towering bulk of disease. But this view fails to comprehend, as is pointed out by the *New York Medical Journal*, the immense number of really effective symptomatic remedies which, in the hands of a sagacious and experienced physician, will bring the storm-tossed vessel safely into port over many a troubled sea. The doctor, like that vigilant muffled figure on the bridge, may not be able to quell the storm, but he can guide and protect, and without such guidance and protection direful shipwreck would be inevitable.

This view also fails to take in what is the better part of medicine, that which prevents disease, and the following brief review will serve to indicate in a measure the benefits that have accrued to man by the application of its principles. Hospital erysipelas and gangrene have been practically eliminated from the list, and the number of cases of wound-infection and blood-poisoning, including child-bed fever, have been enormously decreased. This was largely brought about by the discoveries of Lister and Virchow's dictum that all disease was due to preexisting disease.

In 1800 the average length of life in England was twenty-seven years, in 1900 it was forty-five years, a net gain of 56⅔ per cent.

The London death-rate has been reduced from 5 per cent. to 2 per cent. The death-rate of smallpox has decreased 95 per cent., of fevers in general 82 per cent., of typhoid fever 60 per cent., of scarlet fever 81 per cent., of diphtheria 59 per cent., of tuberculosis 46 per cent.

In 1878 the population of Memphis was 19,500. In that year there were 17,600 cases of yellow fever with 6,000 deaths. Contrast with this the recent outbreak in New Orleans. What city would now tolerate the causes of such a scourge? Prevention has taught that the stagnant cesspool must give way to the ventilated drain, the reeking well and foul cistern must give way to a well-regulated public water-supply.

> The men shall do
> The unknown things, the wondrous deeds
> Earth's future needs
> Demand;
> Its hand
> Shall shape the course,
> Its brain devise
> The plan
> To win the richest prize that man can win—
> The betterment of man.
>
> —*From Press Com. Washington City (Iowa) Med. Society.*

That preventive medicine has done all these things and can do more is sufficient to remove forever the sting of a sneering comment upon the inadequacy, incompetence and inefficiency of the purposes and resources of the medical profession.

